# Anorectal malformation in adulthood: a systematic review of biological, psychological, and sociological outcomes and experiences

**DOI:** 10.1007/s00383-026-06424-4

**Published:** 2026-04-15

**Authors:** Eloise Rane, Julia Bloom, Xi Wen Carys Chan, Paul Harris

**Affiliations:** 1 School of Allied Health, Sport and Social Work, Griffith University, Logan Campus, Meadowbrook, QLD Australia; 2 Department of Management, Griffi th Business School, Griffi th University, Nathan Campus, Nathan, QLD Australia; 3 Centre for Mental Health, School of Human Services and Social Work, Griffi th University, Logan Campus, Meadowbrook, QLD Australia

**Keywords:** Anorectal malformation, Psychosocial functioning, Social work, Psychology

## Abstract

**Purpose:**

Anorectal malformations are congenital anomalies involving the rectum, anus, genital, and urinary tracts, and occur in approximately one in 5000 live births. A lack of transition from pediatric to adult health services means little is known about how this condition is experienced in adulthood. The purpose of this review was to discover biopsychosocial challenges and supports experienced by adults living with anorectal malformation.

**Materials and methods:**

A systematic quantitative literature review was the preferred method to collect and analyze data. Articles included in the review were sorted into purpose-built datasets, with data coded into biopsychosocial themes and patterns.

**Results:**

The most frequently reported biological challenge was surgical intervention; the most frequently reported support was positively framed continence management. The most common psychological challenges were psychosexual concerns such as sexual anxiety, and the most common supports were interventions focused on improving self-perception. The most reported sociological challenge was navigating complex health systems, and the most reported support was forming meaningful relationships.

**Conclusion:**

Biopsychosocial outcomes experienced by adults born with anorectal malformation remain largely unknown. Social work and psychology can provide therapeutic interventions that enhance emotional wellbeing, psychosocial functioning, and patient-led care.

**Supplementary Information:**

The online version contains supplementary material available at 10.1007/s00383-026-06424-4.

## Introduction

### History of ARM

The earliest description of anorectal malformation (ARM) exists in ancient Greek medical writings, whereby Aristotle referenced rectourethral fistula within a recorded but failed attempt to correct the condition via incision [1]. From the early 1980 s onward, surgical intervention for ARM commonly involved the posterior sagittal anorectoplasty (PSARP) and subsequently, existing literature has focused primarily on outcomes related to continence [2]. Following surgery within the newborn period, long-term review and follow-up care is often lost [3] and a lack of transitional care from pediatric to adult health services forms an ongoing barrier to effective management of this condition in adulthood [4]. Therein, the biological, psychological, and sociological outcomes and experiences of living with ARM as an adult have remained largely unexplored, underpinning the importance of this review.

### Etiology and ARM classifications

Whilst the cause of ARM remains unknown, the condition is understood to be multifactorial. Certain risk factors have been indicated to include maternal (Body Mass Index [BMI] ≥ 25 kg/m^2^) prior to pregnancy, maternal fever during the first trimester of pregnancy, paternal exposure to exhaust gases or particulate matter, paternal cigarette smoking, and one or more (first or second relation) family member with the condition [5].

The ‘Wingspread Classification’ was created in 1984 to standardize the assessment of ARM sub-types, surgical work up, and record-keeping of clinical features [6]. The ‘Krickenbeck Classification’ was later formed in 2005 and became the universal clinical guide for ARM diagnoses, allowing experts to compare classifications with improved accuracy [7]. Although the ‘Krickenbeck Classification’ included a recommendation of follow-up care for patients, the recommendation did not exceed ten years post-operatively. This rendered long-term outcomes related to ARM in adulthood potentially being forecast during pediatric care, rather than directly known through follow-up care [8]. The lived experiences and outcomes of adults currently living with the condition therefore required exploration, with the literature base of this review examined on this premise.

## Methods and materials

### Methodological framework

This systematic review aimed to discover, ‘What does the published literature report and prioritize regarding biopsychosocial challenges and supports experienced by adults born with anorectal malformation?’. The biopsychosocial model [9] directly informed the analytical framework of the review. Categories addressed biological (physical symptoms, surgical outcomes), psychological (emotional responses, coping strategies), and sociological factors (interpersonal relationships, healthcare interactions). This approach prevented reductionist tendencies observed in previous research that often prioritized biological outcomes while minimizing psychological and sociological dimensions.

**Fig. 1:**
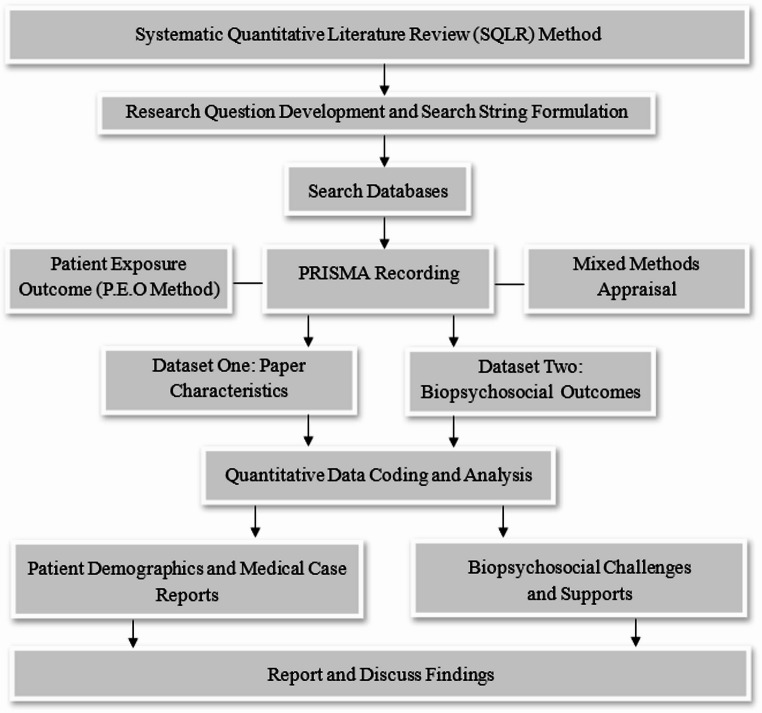
Flow chart of systematic quantitative literature review method

A flow chart representing this process is provided in Fig. 1.

### Eligibility criteria

Inclusion criteria for the systematic review required articles that explored at least one of two broad areas aligned with the research aims. (1) Articles that included an adult ARM population that could be analyzed (2) Articles that reported on expected or experienced biological, psychological, or sociological outcomes in the lives of adults living with ARM. Additionally, articles were required to be peer-reviewed, original research, available in institutional databases, and in full-text English. The search was not limited by publication date, to highlight themes and phenomena over time. Literature reviews were excluded to avoid duplication of data [10]. Information sources included five electronic databases: Google Scholar, Scopus, PubMed, PsycInfo, and CINAHL (Complete via EBSCOhost).

### Search string formulation

Search string formulation remained broad, so as not to skew results towards one domain (biological, psychological, sociological) over another. The Population Exposure Outcome (PEO) method [11] identified the population (adult patients), exposure to a phenomenon (born with anorectal malformation), and outcomes (biopsychosocial). The following search string was employed: (“adult patient*”) AND (anorectal malformation*) and inputted into each database as Database > No limitations applied > Search > All fields > “adult patient*” AND “anorectal malformation*”. No changes were made to settings, limitations, or search fields between databases, enabling reproducibility.

## PRISMA recording

**Fig. 2:**
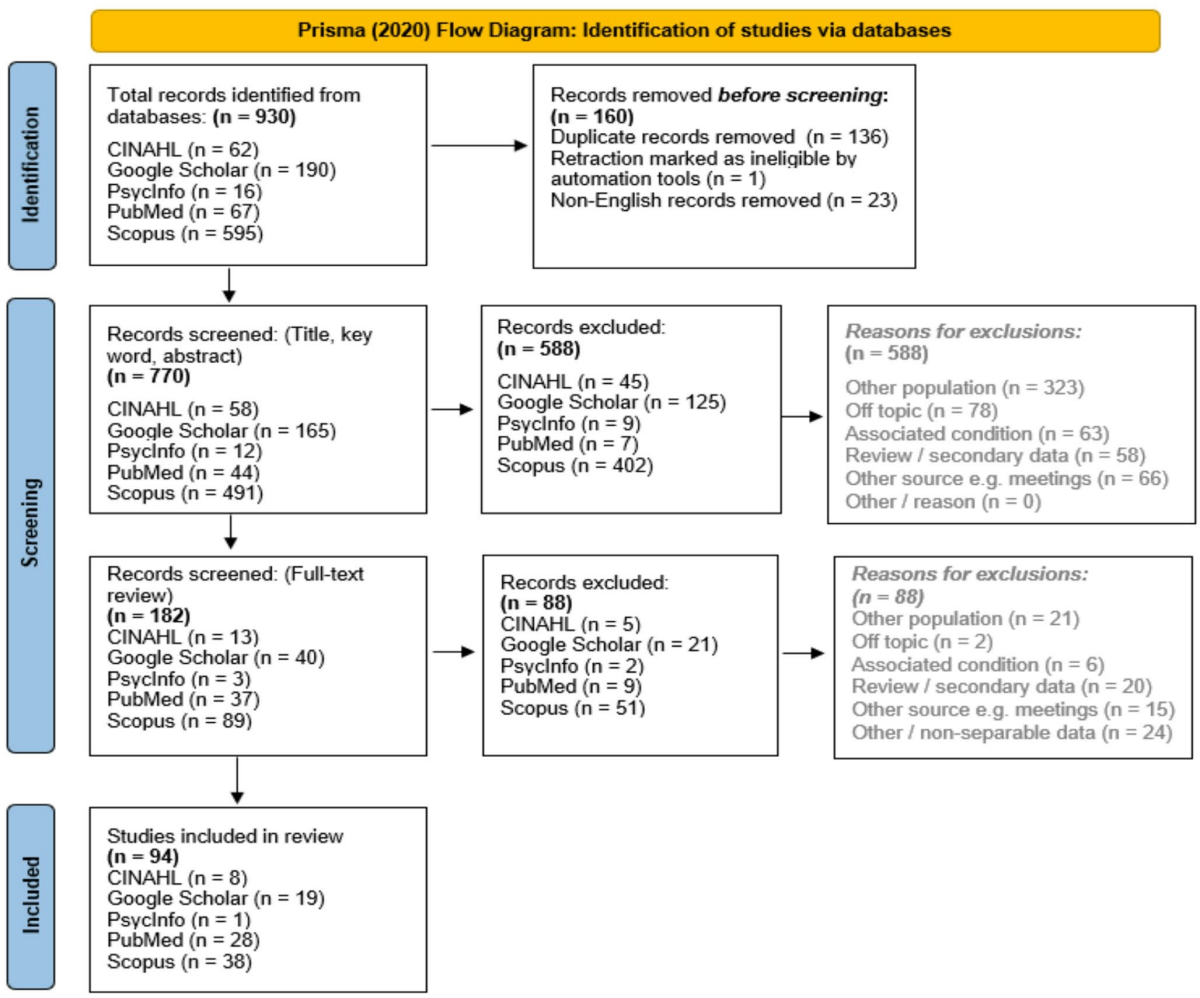
PRISMA (2020) flow chart of literature screening

The Preferred Reporting Items for Systematic Reviews and Meta-Analyses (PRISMA) was the standard used to record screening cycles [12] with results shown in Fig. 2.

### Quality appraisal

Appraisal was completed using the Mixed Methods Appraisal Tool (2018). However, articles were not excluded based on quality appraisal alone, as calculating an overall score is discouraged [13]. Instead, the purpose of conducting the appraisal was exploratory in nature. All articles were feasible for review using the appraisal tool. Only one article utilized a mixed methods approach, five articles were qualitative, and the remaining (*n* = 88) articles were quantitative descriptive. There were no studies reported as quantitative randomized, or non-randomized control trials. The most common methodological quality criteria not met was non-response biases in over half (*n* = 56; 59.5%) of the reviewed articles. This was primarily due to sampling pools and response rates not being provided. In addition to PRISMA recording and quality appraisal, a summary table of the 94 articles included in the systematic review is also provided in Supplementary Table 1.

### Dataset construction

Paper characteristics, authorship, year of publication, population demographics, and medical case reports were recorded in ‘Dataset One’. Biopsychosocial challenges and supports experienced by adults living with ARM were recorded in ‘Dataset Two’, including a sub-section dedicated to transition of care findings. Themes were identified using a block and file approach to data categorization [14], allowing data to be critically examined and include psychological and sociological experiences previously overlooked in literature.

## Results

### Dataset one: paper characteristics

#### Authorship and publication

**Fig. 3:**
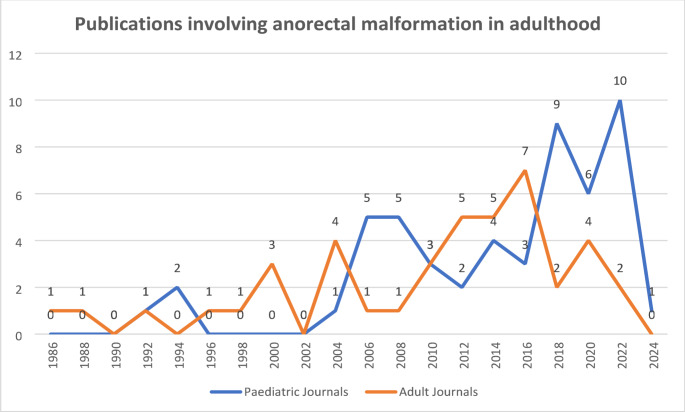
Adult ARM publications in pediatric and adult journals from the year 1986 to 2024

Over half (*n* = 48) of the reviewed articles were published in pediatric journals and most commonly authored by pediatric surgeons, despite reporting on ARM patient outcomes in adulthood. The examined literature spanned 38 years from the year 1986 to the year 2024 with no time limits applied, confirming the systematic approach was appropriate and robust in design. The lowest publication outputs occurred in the 24-month period of 1990 to 1991, and 2002 to 2003 with 0% totals. The highest publication outputs occurred in the 24-month period of 2022 to 2023 at 13%. Publication findings are indicated in Fig. 3.

In addition to the predominance of pediatric authorship and publications, the diagnoses of patients in (*n* = 40) or 42.5% of studies were also reported by the author or surgeon. It therefore cannot be determined if patients represented in those articles were aware of their own diagnoses. Correspondingly, adult ARM patients in (*n* = 20) or 21.2% of studies reported not knowing their diagnosis. In (*n* = 14) or 14.8% of studies, surgical interventions were reported by the author or surgeon, and therefore it could not be determined if patients represented in those articles were aware of surgical interventions they had received. Patients in (*n* = 16) or 17% of articles reported only becoming aware of previous surgical interventions upon requiring corrective surgery in adulthood [15].

#### Population demographics

A total of 3779 adult ARM patients were represented in the reviewed literature base. More than half (*n* = 62) of the included studies were conducted in North America and predominantly within the United States of America. The remining (*n* = 32) studies were conducted in Germany (5), United Kingdom (5), Italy (3), Netherlands (3), Switzerland (2), Japan (2), Denmark (2), and one each for Finland, Czech Republic, Taiwan, Turkey, Singapore, Pakistan, India, Australia, Philippines, and Africa. Patient ages in the dataset spanned from 16 to 71 years of age. Patients in the reviewed studies were found to be an average age of 30.2 years.

#### Medical case reports

Many of the studies included for review were medical case reports (*n* = 25), listed in Supplementary Table 2. While the publication of rare medical cases can assist in furthering medical knowledge, in some instances the inclusion of sensitive imagery and a lack of reporting patient informed consent for those publications was present. Only six of the 25 case reports confirmed they had obtained patients’ consent for publishing; 17 did not discuss whether patients’ consent had been obtained, and two reported that patient consent was not required.

The 25 medical case reports were analyzed through the demographic lens, due to the lack of patient diversity in existing literature [16]. This process uncovered three distinct themes related to how patients from a variety of backgrounds were experiencing the condition. Themes are ordered below by ascending prevalence and year of publication.

#### Theme one: health care avoidance in male ARM patients (16% of medical case reports)

- (2004) Japanese male ARM patient (48 years of age) had colostomy created following birth and surgical revision at age four. However, the malformation was not adequately repaired. As a married adult in his 20’s, he refused surgical repair for imperforate anus (IA) [22].
- (2009) Italian male ARM patient (46 years of age) refused abdominal colostomy and avoided health care for over 40 years despite suffering giant fecaloma on background of anal atresia [26].
- (2015) Japanese male ARM patient (71 years of age) suffered megarectum in later phase of life, speculated by authors to be on background of childhood ARM surgery. This reflected a lack of health service access and intervention for ongoing care [30].
- (2021) Latin American male ARM patient (44 years of age) developed rectal prolapse and bleeding episodes during heavy occupational lifting. The patient did not have health insurance and initially avoided seeking treatment for this reason [39].

#### Theme two: differing impacts on female patients (28% of medical case reports)

- (1989) Finnish female ARM patients (33, 37, and 40 years of age) one patient was diagnosed with ARM at birth, two were not diagnosed until adulthood, and all three patients became bowel incontinent following vaginal birthing deliveries [18].
- (2008) North American female ARM patient (42 years of age) who presented with fecal incontinence. Clinical examination revealed recto vestibular fistula and imperforate anus. PSARP intervention was considered an acceptable option for this patient and helped to dispel the common assumption that adult ARM patients have limited options if not operated on during infancy [25].
- (2009) Indian female ARM patient (17 years of age) whereby authors discussed that ARM patients in developing countries may not present for clinical review or may have their condition incorrectly diagnosed [27].
- (2010) Pakistani female ARM patient (23 years of age) who was diagnosed at two years of age, however, medical care was not followed-up by the parents until the patient was to become married. She was then brought into the hospital for review and reconstructive surgery [28].
- (2017) Turkish female ARM patient (26 years of age) brought to an outpatient clinic by relatives with symptoms of constipation and defection through her vagina. The reasons for delay in presentation were recorded as family negligence, social determinants of poor health, and low education [35].
- (2017) North American female ARM patient (25) reported that ongoing medical care for her condition in adulthood, meant she was able to learn how to perform colostomy flushing independently. The patient found balancing her career improved significantly with self-management [32].
- (2017) Guatemalan female ARM patient (54 years of age) was birthed by unqualified personnel. The patient consulted numerous clinics later in life, for discharge of faeces through her vagina and resulting infections. The lack of access to early intervention resulted in late treatment for this patient, however; surgery in adulthood challenged age as a contradiction for its’ viability [34].
- (2019) North American female ARM patient (30 years of age) whereby a planned caesarean-section (for two consecutive pregnancies) was indicated for reduction of vaginal trauma. Although each pregnancy was complicated by recurrent urinary-tract infections, both caesareans were reported as being straightforward [37].

#### Theme three: the medical model and system deficits (32% of medical case reports)

- (1986) English female ARM patients (17, 20, and 23 years of age) with one patient recorded as Pakistani. All three patients had surgical intervention for ARM shortly after birth, each of which was unsuccessful. The surgeon recorded the attempts to perform anatomical corrections, however, all three patients required acceptance of colostomies. The author detailed one patient’s struggle with dyspareunia associated with penile penetration of the anorectum and indicated that psychosocial impacts relating to the condition had not previously been considered [17].
- (1999) North American female and male ARM patients (mean age 26) received secondary surgical repairs due to persistent incontinence in adulthood. Prior to secondary surgical repairs, all patients had diversion stomas; with one patient retaining their stoma due to secondary repair failure [20].
- (2005) Italian male ARM patient (38 years of age) with anorectal atresia. The unsuccessful installation of a system (artificial bowel sphincter) was reported by authors to be hindered by the medical device being designed for patients with normative anatomical features. Complications included perianal and anal ulceration, system damage, infection, and intervention failure [23].
- (2016) Swiss female ARM patient (27 years of age) who presented with anal canal duplication that was not discovered at birth. Although rare, the author gave indicators for other surgeons to consider, such as any midline opening existing posterior to the anus should raise condition suspicion [31].
- (2017) Taiwanese male ARM patient (55 years of age) who had not received follow-up care after repair of low-ARM, resulting in constipation and mega rectosigmoid. The authors reported that patients with ARM should receive ongoing bowel management as an integral component to surgical intervention; to avoid constipation and improving (QoL) outcomes [33].
- (2019) Batswana female ARM patients (23 and 34 years of age) presented with ill-repair for ARM. Contributing factors included a specialist surgeon only available for three months of the year, and the patient’s location described by authors as resource limited. Additionally, one patient had severe anxiety involving medical trauma and repeated scarring of the anal area [15].
- (2019) Italian female ARM patient (25 years of age) with ectropion of anal mucosa (AME) following ARM surgery. Symptoms of this complication included bleeding, pain, and soiling. The authors reported a complete resolution of symptoms for the patient following secondary surgical intervention. This improved outcome was attributed to a multidisciplinary approach towards evaluation and after care that included a psychologist, stoma therapist, plastic, and pediatric surgeon [36].

These findings highlighted the importance of patient care that considers cultural and contextual factors in the lives of adults with ARM, and the variety of ways in which these elements influence engagement with, or avoidance of health care.

### Dataset two: biopsychosocial challenges and supports

Biological, psychological, and sociological challenges and supports related to living with ARM in adulthood were identified from within the literature base. In the interest of shifting away from deficit-focused paradigms which capture only the challenges, identified supports were also identified and included. Findings are indicated in order of challenges, supports, and theme-prevalence below.

#### Biological challenges

Biological challenges for adult patients living with ARM were observed across six distinct themes from the 94 reviewed studies as follows:

1. Surgical intervention and revisions were identified in (*n* = 24) studies. Examples of these challenges included surgical failure and infection, medical trauma associated with repeated scarring, soiling, pain and bleeding, and inadequately repaired ARM [23, 36, 41].
2. Impacts of fecal incontinence were indicated in (*n* = 21) studies. One such study reported that 33.3% of those participants suffered involuntary urine leakage and 17.4% suffered with stool leakage [41]. One of the most recent studies from 2024 reported that only 28.6% of adult participants had well-preserved continence following pediatric surgery for ARM [42].
3. Sexual dysfunction was reported as an impact related to anatomical difficulties of the condition in (*n* = 14) studies. Examples included maternal morbidities such as urinary infections, vaginal lacerations, clitoral abscesses, and bladder injuries, erectile or ejaculatory dysfunction for male identifying participants, and infertility issues for male and female identifying participants [38, 43, 44].
4. A lack of knowledgeable health care providers and expensive treatment costs were indicated in (*n* = 14) studies. Examples included ongoing surgical debate surrounding the feasibility of intervention in adulthood, high rates of medical trauma observed in the adult population of ARM patients, and low patient engagement for complications associated with managing the condition, such as fecal incontinence [27, 45, 47].
5. Negative impacts of symptoms on the health-related quality of life (HRQoL) of patients were indicated in (*n* = 11) studies. Some symptoms included rectal prolapse, bleeding, and recurrent urinary tract infections [39]. In addition, ARM patients with a stoma, who were older, or female, were reported to experience a poorer (QoL) in adulthood [47, 48].
6. The epidemiology of ARM was an indicated biological challenge in (*n* = 9) studies. One such study reported that the rate at which a child of an adult with ARM may also present with the anomaly as 62% and suggested the condition may operate via an autosomal dominant mode of inheritance [23]. Another study reported that 79% of their participants incorrectly identified their diagnosis type and were therefore unaware of biological outcomes which may relate to their offspring [49]. Only one (*n* = 1) of the 94 studies reviewed did not report on biological challenges experienced by ARM patients in adulthood.

#### Psychological challenges

Psychological challenges experienced by adults living with ARM were identified across six emergent themes in the reviewed literature as follows:

1. Psychosexual challenges were discussed in (*n* = 18) studies and covered a range of patient concerns. Examples included sexual encounters potentially worsening the psychological symptomology of ARM, adults with ARM achieving fewer psychosexual milestones than the general population, and patients with frequent incontinence experiencing higher sexual anxiety [40, 47, 50].
2. A lack of mental health support was indicated in (*n* = 13) studies. Discussions included the need for more paramedical and psychological care, and patients feeling there was ‘no’ mental health support for their condition; including limited professional knowledge regarding the psychological impacts of ARM [47, 51]. One study from 2019 reported that 58% of respondents had a mental health diagnosis [45]. The most common of these being depression 87%, post-traumatic stress disorder (PTSD) 46%, and anxiety 85%. A 2018 study found that one third of all adult ARM patients felt they needed psychological support within the previous two years [52]. However, whether support was provided or accessed remained unclear.
3. Maladaptive coping strategies were an observed theme in (*n* = 11) studies. The most common examples were patient refusal of surgical revision [22, 26], negative self-perception [43], and avoiding follow-up care [53].
4. Psychological functioning was a theme discussed in (*n* = 8) studies. This included patients who had not experienced ‘normal’ bowel behavior, potentially being exposed to serious psychological impacts of the condition; particularly when managing external systems such as stomas [23]. Further examples included medical trauma indicated by severe anxiety during pelvic examinations [15], and data from one study suggested psychological function was impacted by self-perceptions of condition severity [54].
5. Emotional impacts of ARM were discussed in (*n* = 7) studies. These included impaired body image, depression, and feelings of shame, lower levels of emotional functioning in adult ARM patients when compared to pediatric samples, and low levels of emotional well-being and energy [41, 55].
6. Inhibitors on self-efficacy were identified within (*n* = 5) studies as a psychological challenge for adults living with ARM. Patients who felt they were discouraged to take active roles in their health journey during adolescence, often due to parental over-involvement, were found to have difficulty in developing self-efficacy traits essential to navigating the adult phase of the condition [56, 57]. In addition, older surgeons (< 55) were found less likely to consult with adult ARM patients they had treated during childhood than their (> 44) colleagues, despite a lack of adult specialists available for patient transition and impacts on patients developing self-efficacy in managing their condition through adulthood [58]. The remaining (*n* = 32) studies did not report on psychological challenges experienced by adult ARM patients.

#### Sociological challenges

Sociological challenges related to the experience of ARM in adulthood were present across six themes in the literature base as follows:

1. Navigating complex health systems was an identified challenge mentioned in (*n* = 19) of the reviewed studies. Details included changes to terminology, surgical workup, and interventions for ARM reflecting changes in outcomes for patients, and oftentimes, contributed to a lack of patient knowledge regarding their diagnosis [49]. One study from 2022 found that patients are increasingly seeking information related to their condition from online peer support groups, which may indicate a gap of information transference from provider to patient [59]. A 2004 study raised consideration of whether budgeting restraints and treatment costs in health care systems have influenced patient outcomes, such as reducing patient engagement for ongoing management support of their condition [60].
2. Continence and social participation were discussed in (*n* = 15) of the reviewed studies. One such study spoke to the unsatisfactory continence rates amongst the adult ARM cohort, with only 15% of those patients reporting no effects of social disability, and the significant social problems the condition can cause [61]. Another study detailed the significant time, effort, and energy required by patients for the management of their ARM symptoms, such as careful consideration of inputs, outputs, and associated consequences on continence or constipation [33]. A study from 2022 reported continence rates are still not optimal for adults with ARM, with 34.8% of those patients experiencing fecal incontinence and 71.4% experiencing urinary incontinence; significantly influencing perceived choices in social participation [41].
3. Family dynamics were a sociological challenge detected in (*n* = 12) studies. This finding was commonly reported as patient exposure to family negligence of their condition, and low levels of health literacy and education within the family system [35]. Another study found that tensions between parents and ARM patients during childhood can delay sexual development in adolescence and adulthood [62]. For those patients, condition management in childhood such as anal dilations, wash outs, and recurrent UTI’s became associated with pain. In adulthood, this consequently influenced problems with arousal in the genital region.
4. Negative impacts on intimate relationships were indicated in (*n* = 11) studies. One such study found that feelings of demoralization and fatigue contributed to those patients reducing their participation in relationship building; ultimately contributing to depressive episodes [63]. Another example included a marriage wherein the patient reported being unable to complete sexual intercourse [43]. However, impacts of ARM on sexual function remain poorly investigated, and the challenge of poor information provision for patients ensues for adult-specific concerns of intimacy and sexuality [64].
5. Impacts on personal and professional networking were an indicated sociological challenge for patients in (*n* = 10) studies. Examples included patients predominantly opting for sedentary occupations due to their condition, which consequently led to sub-optimal outcomes in social integration [65]. Another study added that networking experiences may also be impacted by the patient’s own personal and social resources or lack thereof; further influencing (QoL) outcomes [64]. Irrespective of etiology, patients from a 2023 study expressed their desire for increased social support and networking groups, to promote social empowerment and information provision related to symptoms and management of condition complexities [66].
6. Impacts on patient resilience were detected in (*n* = 8) of the reviewed studies. Patients whose childhood continence concerns were ignored by parents were found to have problems impacting socialization later in life, such as bowel enema dependence, repeated soiling, involuntary flatulence, and a perceived need for constant toilet access [67]. Juxtaposed, overprotection in childhood by parents also led to a lack of resilience-building opportunities, required when navigating the world with ARM as an adult. One such study discussed the importance of patients developing resilience, particularly when transitioning from pediatric to adult care for their condition. Whereby, disease cognition can become distorted through negative self-perception, becoming a strong adversity that influences (QoL) outcomes for adult patients [47]. The remaining (*n* = 19) studies did not report on sociological challenges.

#### Biological supports

Biological supports reportedly experienced by adults living with ARM were identified across four themes in the reviewed literature as follows:

1. Improved surgical intervention and provider knowledge were indicated in (*n* = 37) of the reviewed studies. Examples included improved preservation of sexual functioning in reconstructive surgeries [55] and improved (QoL) outcomes for patients following graciloplasty transposition surgery using stimulating electrodes [68]. One study also discussed the importance of shifting perceptions of surgical ‘success’ from mere survival, towards observing positive outcomes in the lives of patients [69]. In addition to this shift, improved provider knowledge was attributed to professionals assembling as part of multidisciplinary care teams, rather than operating independently of one another [15]. Examples included holding multidisciplinary meetings to discuss patient concerns [57], through to ensuring basic care support and knowledge regarding the condition could be provided by family practitioners [51].
2. Effective bowel management in adult ARM patients was a support theme indicated in (*n* = 24) studies. One such study reported that 81% of patients were satisfied with their bowel management plans [70]. However, another study reiterated the importance of providers understanding a ‘good’ outcome of effective bowel management, was still not a clear indicator of ‘normal’ anorectal function; with this distinction remaining imperative to ongoing care still being a requirement for ARM patients [67].
3. Positive sexual functioning was a theme indicated in (*n* = 13) studies and was discussed across a range of results. One study reported that erectile and sexual dysfunction were unaffected by the presence of spinal defects commonly associated with the condition [64]. Another study found that the severity of the ARM-type was not inherently correlated to poor sexual functioning [71]. Female patients reportedly experienced improvements in sexual functioning through active information-seeking with their gynecologists [66]. And another study discussed the importance of information provision taking place between provider and patient, rather than parent and patient, preferably before the onset of puberty; being essential to positive sexual outcomes for adult patients with ARM [53].
4. Positive coping strategies were indicated in (*n* = 10) of the reviewed studies. Examples included patient networks for information sharing and symptom management, and coping mechanisms that focused on active support seeking and planning [59, 72]. Genetic counselling was also discussed in one study, whereby improved patient understanding led to positive coping around ARM occurrence rates in family planning [72]. The remaining (*n* = 10) articles did not report or indicate biological supports experienced by adult patients living with ARM.

#### Psychological supports

Psychological supports were identified across four themes from the reviewed literature base as follows:

1. Psychological interventions for adults living with ARM were recommended in (*n* = 13) studies. Examples included interventions efforts that focus on psychological treatment for body image and emotional distress [74]. One article reported that cognitive behavioral therapy (CBT) and talk therapy were the most common intervention recommendations offered to patients at 51% and 82% respectively [45]. Additionally, one study recommended health care professionals adopt a multidisciplinary approach to patient evaluation, specifically to include psychological services [36].
2. Independence stimulators were an evident theme within (*n* = 7) studies. Strategies that reduced self-blame, substance abuse, behavioral disengagement, and denial were found to enhance patients’ independence and overall HRQoL [72]. Another study discussed the higher rate of university enrolment amongst adult ARM patients compared to a general population sample [65]. Suggesting the need to become more independent at a younger age resulted in a greater sense of discipline and improved ability to prioritize independence. Patient participation in online peer support groups also established independent access to health information and reportedly improved individuals’ mental health and wellbeing [59].
3. Age-appropriate health literacy was an identified theme discussed in (*n* = 7) of the reviewed studies. Examples included ensuring that information sharing, management, and preservation of sexual functioning was closely aligned with corresponding psychosexual development [69]. This could be achieved by health care professionals considering developmentally appropriate timing for discussions with patients around sexual and reproductive health [66]. One study reported 96% of patients feeling that practitioner development of ‘soft skills’ and ‘professional knowledge’ were very important to them when discussing their condition and concerns in health care settings [52].
4. Patient empowerment was an identified psychological support identified in (*n* = 6) studies. Findings indicated psychosocial supports such as everyday connections, companionship, esteem-building, and shared problem-solving enhanced patients’ (QoL) and psychosocial functioning over time [68]. Adult ARM patients who felt more empowered about their body felt less embarrassment and shame about their physical condition [71]. Importantly, adult patients who felt psychologically empowered sought earlier follow-up care for their condition and reported positive impacts in their lives, such as the desire to spend more quality time with their family and children [39]. The remaining (*n* = 61) articles did not report on psychological support for adults living with ARM, composing the largest research gap in the reviewed literature.

#### Sociological supports

Sociological supports in the lives of adults with ARM were observed across four themes within the reviewed literature as follows:

1. Establishing close or intimate relationships was an indicator of positive support construction in (*n* = 16) studies. Positive sexual and intimate relationships were considered supportive factors [51, 55]. Harmonious family dynamics were also a significant determinant of support associated with higher levels of psychosocial functioning [75]. One study discussed the importance of patient sexual health and sexuality not being solely attributed to the absence of dysfunction, but rather, a state of physical, emotional, mental, and social wellbeing [76].
2. Social participation and networking was an identified support in (*n* = 14) studies. This theme was indicated in reports of patients actively seeking social and emotional support from their networks, reframing acceptance positively, and turning to spirituality-based systems such as religion for social participation and support [73]. Surgeons who participated in nationwide registers aimed at supporting ARM more comprehensively contributed to networking opportunities for patient concerns [76].
3. Optimism for life was a support indicated in (*n* = 14) studies. Examples included patients who held optimistic views for their life, self, and future, partially mediated the mental and physical effects of symptoms such as fecal incontinence impacting social participation [77]. In one study, a patient shared their experience of improved occupational outcomes, in the hope their story may prove useful in optimizing the outlook for others navigating ARM in adulthood [39].
4. The achievement of developmental milestones was a theme observed across (*n* = 13) studies. Appropriate levels of parental involvement in health care settings during adolescence were attributed to improved follow-up care by patients during adulthood, including a deep appreciation for that involvement [57, 67]. Another example discussed the importance of patients developing communication skills necessary to navigate their condition in both health and social settings [54]. The remaining (*n* = 37) studies did not report on sociological supports which may mitigate the challenges experienced by adults living with ARM.

**Fig. 4:**
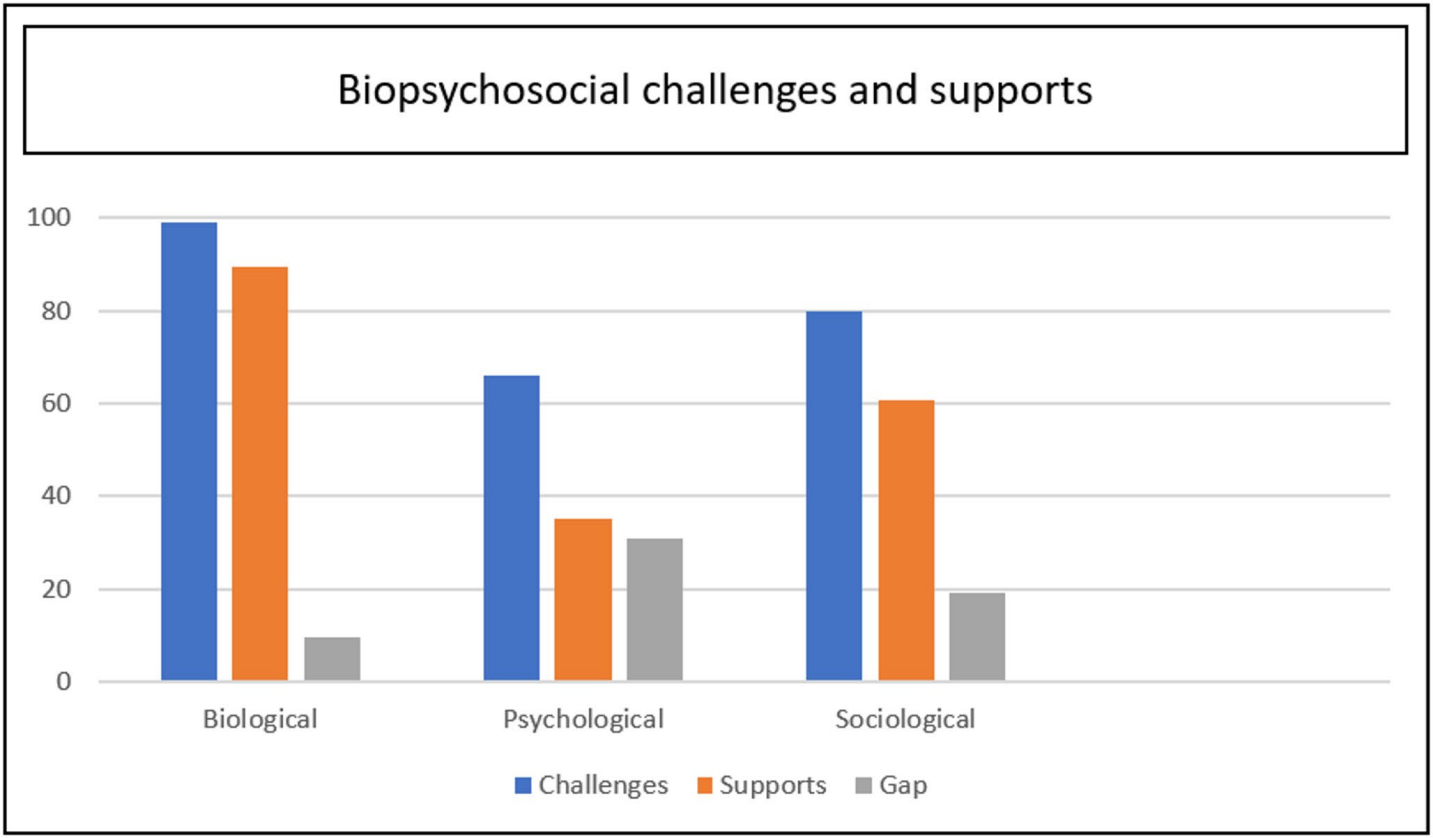
Percentages of biopsychosocial challenges and supports from the literature base

When combined, biological challenges for adults with ARM represented 98.9% of reported experiences or outcomes prioritized in the literature base. Biological supports equalled 89.3% of reporting. Psychological challenges were indicated in 65.6% of the literature, with psychological supports indicated in only 35.1% of the literature. Sociological challenges were discussed in 79.7% of the literature, and sociological supports in 60.6%. These findings demonstrate quantified experiences and outcomes, producing overall percentages of each domain (bio-psycho-social). Figure 4 provides a visual representation of the results below.

Results additionally demonstrated the gap between identified challenges and supports. Biological challenges for adults living with ARM exceeded identified supports by 9.5%, indicating bio-medical outcomes are the most strongly represented in the literature base. Psychological challenges exceeded supports by 30.8%, making it the least mitigated challenge of the three domains. Sociological challenges exceeded supports by 19.1%. Demonstrating this domain also requires increased intervention and support for adults navigating life with this complex condition.

### Transitional and follow-up care

A sub-category of data from the 94 reviewed studies were collected during coding and analysis, relating specifically to transition of care and follow-up care outcomes for adult patients with ARM. It was of importance that this data be recognized, analyzed, and reported, due to the influence of transitional care from pediatric to adult health services on future engagement and effective management of a lifelong condition. A combined (*n* = 13) studies discussed transition of care modalities for patients with ARM. Interestingly, reporting did not discuss whether patients had completed a transition of care between pediatric and adult services, but rather, provided recommendations for this to occur as follows:

- Implement a ‘transition period’ [78].
- Conduct more research regarding the needs of ARM patients as they transition into adulthood, and what expected outcomes of the condition might be [79].
- Provide more structured follow-up processes for adult ARM patients [46].
- Experienced providers are needed [66].

Follow-up care was reportedly received by patients in a total of (*n* = 19) studies.

However, data available for further analysis indicated a range of factors with significant implications for adult patients as follows:

- Follow-up care was not received beyond four years of age [22].
- Follow-up care was received but remained in pediatric settings [80].
- Follow up care was received but there was a reportedly low level of patient understanding [54].
- Follow-up care was received by only three out of 68 patients in the study [65].
- Follow-up care continued but only by pediatric surgeons [58].
- Follow-up care at its’ longest point reached 20 to 25 years of age [81].
- Follow-up care was received by patients, but did not exceed 23 years of age [42, 82].

Such examples demonstrated the importance of not only collecting and categorizing data from the literature base but also performing detailed data analysis when exploring contextual factors tied to reported outcomes. A further (*n* = 29) studies reported that no transition of care or follow-up care was received by those patients. The remaining (*n* = 33) studies did not report on whether or not transition of care or follow-up care had occurred.

## Discussion

Being born with anorectal malformation is a rare human experience, and the outcomes related to living with this condition in adulthood remain significantly unexplored. The majority of extant literature is authored by pediatric surgeons. This may suggest the lack of transitional care faced by patients, is also faced by pediatric surgeons; with health professionals knowledgeable on the condition remaining limited in adulthood. Incomplete medical records, alongside unknown surgical histories from childhood, leaves many adults living with ARM without autonomy over their own body and health journey. This in turn can contribute to difficulties in self-advocacy and avoidance in future health care engagement and participation.

Patients within the reviewed literature were found to be an average age of 30.2 years old. This indicates adult individuals with ARM are returning to surgeons for clinical review long after the Krickenbeck (2005) recommendation of 10-years post-operatively. Studies focused primarily on continence outcomes, with authors indicating long-term continence outcomes using cross-sectional data acquired during the pediatric phase of development. Thereby forecasting outcomes for adulthood from observations and assessments conducted during pediatric involvement [34]. In this regard, previous research approaches have remained somewhat reductionist in failing to capture the phenomenological complexities of the human experience of anorectal malformation and adulthood, which potentially differs from childhood experiences of the condition.

Over a quarter of the studies included for review were medical case reports, the majority of which did not report, obtain, or felt it was not necessary to obtain patient informed consent to publish sensitive information or images. This finding highlights the importance of patient-led care that includes the right to autonomy over one’s own body. Particularly within a population that oftentimes had this right distorted through childhood experiences in medical settings. Strong themes from within the medical case reports suggested that in adulthood, health care avoidance in male ARM patients may be due to a range of factors, such as adaptation to their condition and circumstances, societal stigmatization of the condition, and low levels of patient health literacy [21].

However, female ARM patients displayed frequent and positive help-seeking behaviors towards healthcare access for their condition but were oftentimes negatively impacted by socio-cultural influences that resulted in delayed presentation or clinical review. Limited studies discussed protocols for childbirth delivery modes; however, a distinction was observed between outcomes for women with a history of ARM birthing children in resource-limited environments that suffered total bowel incontinence [34]. Differing from women with a history of ARM birthing children in developed nations reporting adequate access and support in childbirth [37]. In this regard, it was found that a multi-disciplinary approach to management should include but is not limited to pediatric, urology, and gynecology as best practice to provide evaluation and surgical work-up for adult female ARM patients [35]. Differing surgical techniques and classifications across time were provided as rationale for difficulty in comparing continence outcomes of adult ARM patients [20] however, despite this difficulty; authors continued to utilize continence-based measures as the primary driver of how studies were orientated. How adult patients were experiencing, coping, or not coping with their condition remained significantly unexplored in this manner; highlighting the importance of collecting paper characteristics and biopsychosocial data for analysis.

Only one study from the 94 included for review did not report on biological challenges associated with ARM in adulthood, further indicating the high prevalence of biologically focused inquiries in the extant literature. In contrast, (*n* = 32) studies did not report on psychological challenges experienced by adult ARM patients; demonstrating the significant gap in literature prioritizing this important aspect of the condition. Sociological challenges were not indicated in (*n* = 19) of the reviewed studies, despite little being known about how adults are navigating this condition in social and occupational settings.

Only (*n* = 10) of the reviewed studies did not report on biological supports, which may be an indication of literature prioritizing outcomes of perceived bowel-continence, with associated surgical innovations and achievements. In contrast, (*n* = 61) studies did not report on psychological supports that may be helpful for adult individuals with ARM. This was despite the literature increasingly indicating mental health concerns that adjoin the condition such as anxiety and depression. A further (*n* = 37) studies did not report on sociological supports in the lives of adults with ARM. Psychosocial experiences and outcomes of support therefore remained less prioritized than biological outcomes in the reviewed literature; despite the needs of adult patients with ARM inclined to differ significantly from outcome measures employed during the pediatric phase of management.

Lastly, a distinct lack of transition of care and follow-up care was indicated in the reviewed literature base. This highlighted the need for longitudinal research, the collection of primary data, and qualitative approaches to amplify the voices of patients [35]. First-hand accounts are needed to gain a consensus from the adult cohort in particular, as to the concerns they would like prioritized in ongoing management and support. Distinctly, these needs will require researchers and practitioners to involve an acknowledgement of the complex mental and emotional impacts which so often adjoin this lifelong condition, in order to address the gap in psychosocial understanding observed in the literature.

### Potential research limitations

Studies included in this systematic quantitative literature review were required to be available in full-text English, due to the author’s own language being English. Therefore, studies could not be reviewed if published in a language other than English. To help mitigate this limitation, all discovered studies (including those not published in English) were still included in the process of PRISMA recording and screening. This ensured the existence of those authors’ works was recorded, despite not being eligible for inclusion in the review beyond PRISMA recording and screening. Other authors may elect to remove non-English articles prior to exportation, however, this study elected to ensure the works of non-English authors were represented in PRISMA recording.

The systematic review process is also designed to be reproducible. When inputting data into categories, authors are required to select the corresponding domain; which can impact discussions on discovered patterns and themes. An example from this review were the biological, psychological, and sociological domains; which required decision-making in allocating an outcome or experience to the most appropriate category. Despite this limitation, the systematic review method was reported in full detail, including a visual flow chart and PRISMA recording. This provided a transparent study that aimed to generate future research interest on this important topic.

## Conclusion

Findings from the systematic review indicated that more than half of the literature on anorectal malformation outcomes and experiences in adulthood were still authored by pediatric surgeons. Patient demographics were commonly underreported, with elements of diversity often unexplored as a factor in the lives of patients that influenced how they address their health care needs. Medical case reports represented just over a quarter of the reviewed literature. These reports offered unique insights into individual experiences of ARM in adulthood but also demonstrated inconsistencies in reporting whether or not patient informed consent had been obtained. Male identifying ARM individuals were often found to avoid health care access for their condition and symptom management, while female identifying ARM individuals were found to display positive help-seeking behaviors towards accessing medical literacy about their condition and concerns. Male and female identifying ARM individuals were most commonly requiring clinical review at around age 30. This indicated that the Krickenbeck Classification (2005) recommendation of a 10-year post-operative follow-up may require extension. Improved transition of care and follow-up care models are also needed in order to support patients who have survived through to adulthood due to advancements in surgical techniques across time.

Biological challenges faced by adult patients with ARM were most strongly indicated through stressors of surgery and surgical revision. However, biological challenges were also the most prioritized by the literature. Improved provider knowledge was consistently reported as an outcome which mitigated biological challenges for patients. Psychological challenges most strongly related to psychosexual anxiety and depressive episodes and were the least prioritized by the literature. This demonstrated a significant lack of support and therapeutic intervention for those patients, despite the increasing awareness of mental health diagnoses and concerns which adjoin the condition. Sociological challenges such navigating complex health systems were somewhat met with supports. The most helpful support being patients who developed strong intimate and social connections; particularly those that enabled peer-to-peer information sharing regarding their condition.

Psychosocial support and intervention strategies can be employed by allied health professionals such as social workers and psychologists within multidisciplinary teams to support adult patients with ARM, particularly during their engagement with complex health systems. Limited research currently investigates the importance of this concept, despite the reported psychological impacts of the condition and lack of follow-up care in adulthood. The adult ARM cohort can offer invaluable and nuanced perspectives on what they require from health professionals in order to effectively navigate not only the physical demands of this condition, but also the significant psychological and social aspects. Ongoing research should therefore prioritize first-hand accounts of ARM outcomes and experiences which are best understood by those living with it.

## Supplementary Information

Below is the link to the electronic supplementary material.

Supplementary Material 1

Supplementary Material 2

## Data Availability

All data supporting the findings of this study are available within the paper.
