# Supplementary material for: Anorectal malformation in adulthood: a systematic review of biological, psychological, and sociological outcomes and experiences
Source: Pediatr Surg Int. 2026 Apr 15;42(1):191. doi: 10.1007/s00383-026-06424-4 (PMC13083316; doi:10.1007/s00383-026-06424-4)
Supplement: Supplementary file 1 — Supplementary Material 1 [file 383_2026_6424_MOESM1_ESM.docx]

*Table 1.* Summary table of 94 articles included in the systematic review
