# Supplementary material for: Anorectal malformation in adulthood: a systematic review of biological, psychological, and sociological outcomes and experiences
Source: Pediatr Surg Int. 2026 Apr 15;42(1):191. doi: 10.1007/s00383-026-06424-4 (PMC13083316; doi:10.1007/s00383-026-06424-4)
Supplement: Supplementary file 2 — Supplementary Material 2 [file 383_2026_6424_MOESM2_ESM.docx]

| **Reference** | **Author** | **Year** | **Discussed** |
| --- | --- | --- | --- |
| 1. [17] | Keighley et al., | 1986 | Re‐routing procedures for ectopic anus in the adult |
| 2. [18] | Rintala et al., | 1989 | Surgical repair of vulvar anus in adults |
| 3. [19] | Simmang et al., | 1997 | Posterior sagittal anorectoplasty: primary repair of a rectovaginal fistula in an adult |
| 4. [20] | Simmang et al., | 1999 | Posterior sagittal anorectoplasty in adults: secondary repair for persistent incontinence in patients with anorectal malformations |
| 5. [21] | Violi et al., | 2001 | Rectal cancer in anorectal malformation with rectovestibular fistula |
| 6. [22] | Odaka et al., | 2004 | Anorectal agenesis with a rectourethral fistula diagnosed in an adult: report of a case |
| 7. [23] | Bracale et al., | 2005 | Anorectal atresia treated with non-continent pull through and artificial bowel sphincter: a case report |
| 8. [24] | Hohlschneider & Hustson | 2006 | Adult sexual function after anorectal malformation repair |
| 9. [25] | Shaaban & Heise | 2008 | Posterior sagittal anorectoplasty for congenital rectovaginal malformations in the adult |
| 10. [26] | Altomare et al., | 2009 | Giant fecaloma in an adult with severe anal stricture caused by anal imperforation treated by proctocolectomy and ileostomy: report of a case |
| 11. [27] | Chakravatty et al., | 2009 | Successful management in neglected cases of adult anorectal malformation |
| 12. [28] | Bokhari et al., | 2010 | Late presentation of a patient with an anorectal malformation (ARM) |
| 13. [16] | Miglani et al., | 2012 | Anorectal anomalies in adults: laparoscopic management and review of literature |
| 14. [29] | Thomas et al., | 2013 | Sacral nerve stimulation for fecal incontinence secondary to congenital imperforate anus |
| 15. [30] | Nakayama et al., | 2015 | Postoperative megarectum in an adult patient with imperforate anus and rectourethral fistula |
| 16. [31] | Gülen et al., | 2016 | Anal canal duplication in adults: report of five cases |
| 17. [32] | Brunner et al., | 2017 | Sacral nerve stimulation for fecal incontinence in patients with sacral malformation |
| 18. [33] | Hsu et al., | 2017 | Intractable constipation in an adult with mega rectosigmoid following repair of low-type anorectal malformation |
| 19. [34] | López et al., | 2017 | Anorectal malformations: definitive surgery during adulthood |
| 20. [35] | Tavusbay et al., | 2017 | Successful management without protective colostomy in an adult patient with anorectal malformation |
| 21. [15] | Bedada et al., | 2019 | Managing adults with previously treated anorectal malformations |
| 22. [36] | La Pergola et al., | 2019 | VY anoplasty for ectropion of anal mucosa in an adult with anorectal malformation |
| 23. [37] | Walsh et al., | 2019 | Two successful pregnancies after a previous cloacal repair |
| 24. [38] | Kawaguchi et al., | 2021 | Pregnancy outcomes in 2 women born with complex anorectal malformations: challenges and considerations |
| 25. [39] | McShane et al., | 2021 | Adult residual rectourethral fistula and diverticulum presenting decades after imperforate anus repair: a case report |

*Table 2.* Medical case reports captured in the systematic review
